# Metabolic Switch in the Tumor Microenvironment Determines Immune Responses to Anti-cancer Therapy

**DOI:** 10.3389/fonc.2018.00284

**Published:** 2018-08-13

**Authors:** Barbara Wegiel, Marta Vuerich, Saeed Daneshmandi, Pankaj Seth

**Affiliations:** ^1^Department of Surgery, Beth Israel Deaconess Medical Center, Boston, MA, United States; ^2^Cancer Research Institute, Beth Israel Deaconess Medical Center, Boston, MA, United States; ^3^Division of Interdisciplinary Medicine, Beth Israel Deaconess Medical Center, Boston, MA, United States; ^4^Department of Medicine, Beth Israel Deaconess Medical Center, Boston, MA, United States

**Keywords:** tumor microenvironment (TME), metabolic reprogramming, immune stroma, HIF-1α, lactate

## Abstract

Tumor-induced immune tolerance permits growth and spread of malignant cells. Cancer cells have strong influence on surrounding cells and shape the hypoxic tumor microenvironment (TME) facilitating cancer progression. A dynamic change in glucose metabolism occurring in cancer cells and its influence on the TME are still poorly understood. Indeed, cancer and/or immune cells undergo rapid adaptation in metabolic pathways during cancer progression. Metabolic reprograming affects macrophages, T cells, and myeloid derived suppressor cells (MDSCs) among other immune cells. Their role in the TME depends on a nature and concentration of factors, such as cytokines, reactive oxygen species (ROS), growth factors, and most importantly, diffusible metabolites (i.e., lactate). Further, the amounts of available nutrients and oxygen as well as activity of microbiota may influence metabolic pathways in the TME. The roles of metabolites in regulating of the interaction between immune and cancer cell are highlighted in this review. Targeting metabolic reprogramming or signaling pathways controlling cell metabolism in the TME might be a potential strategy for anti-cancer therapy alone or in combination with current immunotherapies.

## Introduction

### Metabolic switches in the TME during cancer progression and responses to therapy

Majority of cancers show complex metamorphoses in metabolism and composition of immune stroma during their progression (1, 2). This includes metabolic switch toward Warburg biology, which is associated with immune suppression. Therefore, understanding of the molecular mechanisms and signaling pathways regulating metabolism in the TME is crucial for developing novel therapies. Among current anti-cancer approaches, immunotherapy targets the immune cells in the TME and has been successfully applied for subset of patients with melanoma or lung cancer. However, only a small percentage of patients are fully responsive to this regimen. We and others propose that the therapeutic responses are partly dependent on the metabolic status of immune and cancer cells in TME (3–6). In early stages of the disease, immune cells suppress tumor growth, but as tumor becomes established, they favor cancer progression (7) and resistance to the therapy (8, 9). Here, we focus on describing metabolic switches in immune cells in the TME during cancer progression as the targets of future therapies.

#### Role of metabolic switch in tumor-associated macrophages (TAMs) during cancer progression

**Figure 1:**
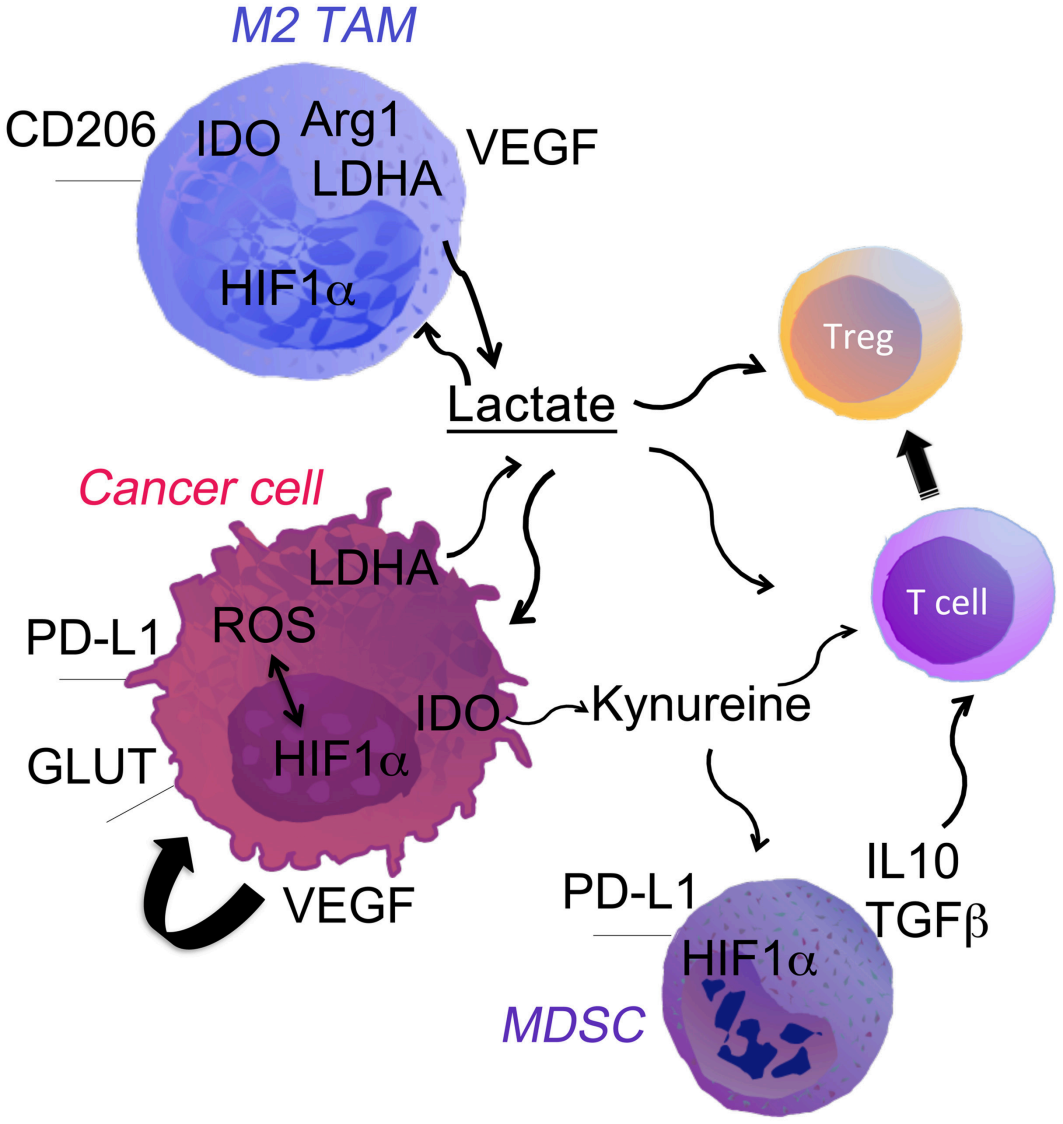
Cell fate in the hypoxic TME. Low oxygen levels, typical of the poorly vascularized tumor milieu, can affect both tumor and immune cells through the stabilization of HIF-1α. It directly promotes tumor growth through the upregulation of genes involved in glycolysis, LDH-A. HIF-1α up-regulation drives expression of PDL-1 and Arg-1 that potentiate the immune suppressive TME. Hypoxia-induced HIF1α and LDH-A-derived lactate strongly modulates the TME. Lactate acts directly on the cells or through the pH changes in the niche. The most notable effects are: (i) polarization of TAM into M2 macrophages; (ii) accumulation of myeloid-derived suppressor cells (MDSCs) and T regs; (iii) inhibition of T effector cells.

TAMs are one of the main cells that define cancer development and progression (10, 11). TAMs are immunosuppressive and polarized toward an M2 phenotype in established tumors. M2-polarized TAMs promote angiogenesis and tissues remodeling. However, in some patients accumulation of TAMs in the TME was associated with better prognosis. Indeed, mice inoculated with tumors with high number of TAMs present in the niche at the time of initiation had smaller tumors (12). Further, Sica et al showed that recruitment of myeloid cells from the blood and their subsequent differentiation toward TAMs may lead to cancer regression (11). ROS and reactive nitrogen species (RNS) generated by TAMs can induce cancer cell death leading to tumor regression or genomic instability supporting malignant transformation. Moreover, M2-polarized TAMs suppress adoptive immunity as well as release of growth factors and matrix proteinases that block tumor progression (13). Cancer cells dictate differentiation and polarization of TAMs into an M2 phenotype via regulation of metabolic switches in the TME. Recent studies show the spatial organization of TAMs populations based on the hypoxia and lactate levels in the TME (14). Indeed, hypoxia promotes M2 like phenotype of TAMs characterized by low MHC-II (15). TAMs with high arginase-1 (Arg-1), and mannose receptor C type 1 (CD206) accumulate in the regions with lower blood and oxygen supply (Figure 1). In these regions TAMs express hypoxia responsive factor-1α (HIF-1α) and switch their metabolism to glycolytic fermentation. In the TME, TAMs are exposed to particularly high concentration of cancer cell-derived lactic acid. Intriguing, the lactic acid stabilizes HIF-1α in TAM under hypoxic as well as normoxic conditions. Lactic acid induces polarization toward M2-like TAMs, which are characterized by higher Arginase-1 (Arg-1) and VEGF levels (16) (Figure 1). Thus, cancer cells induce vicious cycle by generating lactate that further promotes acidic pH by activation of hypoxic responses in TAMs. Moreover, in response to lactic acid, TAMs produce high levels of anti-inflammatory cytokines (i.e., IL-10), which are immunosuppressive. We have recently shown that deletion of lactate dehydrogenase A (LDH-A) and subsequently lactate in myeloid cells leads to regression of lung cancer and is associated with stronger anti-cancer immune responses (6). These findings demonstrate extensive influence of TME metabolic reprogramming of TAMs away from M2 polarization.

Multiple metabolic genes have been implicated in regulation of Mϕ polarization and control of TAMs phenotype (17). As an example, the tumor pyruvate kinase PKM2 inhibits LPS-induced IL-1β secretion, and thus limits the M1 phenotype (18). Further, O'Neill et al showed that PKM2 blockade suppressed PDL-1 expression on TAMs, dendritic cells, T cells and tumor cells (19). Such regulation occurs via direct binding of PKM2 and HIF1α to the PDL-1 promoter. Interestingly, we found that LDH-A/lactate similarly promoted PDL-1 expression in the TME (6). Although, the mechanism of lactate-induced PDL-1 expression is not known, it is possible that such regulation involves HIF1α (20) In addition to glycolytic switch, resting Mϕ or that present the TME are highly dependent on the glutamine-glutamate pathway (21). TAMs express high levels of the glutaminase, which is involved in the glutamine metabolism. Glutaminolysis is important for polarization of Mϕ to M2 phenotype by inducing high α-ketoglutarate/succinate ratio in the TME (22).

#### Metabolic pathways controlling lymphocytes

T cells, B cells, and Natural Killer (NK) cells play a pivotal role in anti-cancer responses (23). In order to destroy cancer cells, effector T cells undergo activation followed by rapid proliferation. These cells are heavily dependent on the supply of oxygen and metabolites, which is limited in the TME. Hypoxia and low nutrient content change T cell metabolism and their anti-cancer responses. In such conditions T cells rapidly switch from oxidative phosphorylation to glycolysis (24). Simultaneously, these cells are exposed to extracellular lactic acid from the neighboring cells that strongly inhibits T cell expansion (25). Of note, naïve T cells depend on energy from fatty acid oxidation (FAO), oxidative phosphorylation, and glutamine metabolism; upon activation, T cells require more nutrients and rely on glucose and glutamine metabolism (26). Changes in lipid metabolism, are also implicated in modulation of CD8+ T-cell anti-tumor immunity. Inhibition of acetyl CoA acetyl transferase 1 (ACAT1) results in enhanced effector functions of CD8+ T cells. Recent publication by Patsoukis et al. highlights the role of PD-1 in T-cell development by promoting FAO via carnitine palmitoyltransferase (CPT1A) (26). Several compounds, such as inhibitor of lipogenic enzymes, have already been tested in preclinical models of cancer. This includes recent combinatorial studies with anti-PD-1 and ACAT1 inhibitor showing positive results in mouse melanoma (27, 28). Avasimibe, an ACAT1 inhibitor, is an established drug for atherosclerosis with a good human safety profile.

#### Myeloid derived suppressor cells (MDSCs) depend on amino acids and fatty acids metabolism

MDSCs are part of the bone marrow derived cell population that accumulates under inflammatory conditions and in the TME (29). Human MDSCs are defined by expression of the alpha M-integrin CD11b and myeloid (CD14 and CD33) or granulocyte/neutrophil (CD15) markers. Murine MDSCs are characterized by simultaneous expression of Gr-1 and CD11b. MDSCs accumulate in the TME and strongly limit the anti-cancer immunity. MDSCs produce immunosuppressive cytokines such as IL-10 and TGF-β, which drive expansion of regulatory T cells and suppression of NK cells. The suppressive activity of MDSCs is strongly regulated by oxidative stress and amino acid metabolism (30). The depletion of important amino acids, like L-arginine, and L-tryptophan inhibit T cells proliferation and increase kynurenine. Kynurenine—a tryptophan metabolite—induces generation of T regulatory cells (24, 31). MDSCs rely on FAO, which is a major metabolic fuel for production of inhibitory cytokines. Thus, targeting of FAO is a possible strategy to limit MDSCs suppressive function. Raloxizine, a FDA approved FAO inhibitor blocked MSDCs expansion and functions in immunogenic preclinical models (32).

### Metabolic reprogramming of cancer cells as a target of anti-cancer therapies

#### Warburg effect and glucose metabolism in cancer cells

Proliferating tumor cells convert most of the glucose into lactate, even in highly oxygenated environments. This aerobic form of glycolysis is described as “Warburg effect” (33). We now know that the Warburg effect is a key feature of all hypoxic cells as well as highly proliferating cells, such as activated effector T cells (34). In contrary to the Warburg's suggestion, most of cancer cells do not have defects in mitochondria or oxidative phosphorylation (34, 35). Metabolic switch to fermentative glycolysis in cancer cells are triggered by various factors that favor tumor survival in hypoxic condition (Figure 1). Hypoxia is accompanied by increased in ROS. High-energy consuming processes including protein, DNA, and fatty acid synthesis are also accompanied by increase in ROS levels (36–39). Cancer cells produce high levels of ROS and are more susceptible to oxidative stress. Cytotoxic anticancer drugs that stimulate H_2_O_2_ production in cancer cells can induce tumor cell apoptosis (38). These treatments also select more aggressive cancer cell clones that have repressed mitochondrial activity (40) and higher ability to survive in hypoxic niche.

#### Glutamine metabolism in cancer cells

Although not classified as an essential amino acid, under conditions of high energy demand, glutamine is a growth-limiting nutrient for cancer cells. A competition for glutamine between cancer cells and MDSC may take place as glutaminolysis supports MDSC maturation (41). The process of glutaminolysis, catalyzed by mitochondrial enzyme glutaminase, leads to conversion of glutamine into glutamate. Glutamate, is further converted into α-ketoglutarate which enters to the TCA, where it serves as a substrate for fatty acids, amino acids, and nucleotides syntheses. This process is controlled by growth factors and oncogenes (i.e., c-myc) (42). Additionally, glutamine can be converted into glutathione, which controls redox state balance. Glutaminolytic rate is considerably higher in cancer cells compare to normal non-malignant cells. Therefore, glutaminolysis has been identified as a potential vulnerability in cancer cells and is an important pharmaceutical target for anti-cancer drugs (43).

#### Lipid metabolism in cancer cells

Metabolic reprogramming in cancer cells due to high energy demands affects lipid synthesis. Cancer cells upregulate monoacylglycerol lipase, acetyl-CoA carboxylase (ACC), and fatty acid synthase (FASN), all which are involved in lipid metabolism. Increased expression of ATP citrate lyase (ACLY), which promotes cholesterol synthesis, is also a common feature of several tumors (44). Interestingly, neoplastic cells can acquire additional energy source from nearby adipocytes. Metastatic ovarian cancers cells convert triglycerides (TGs) from adipocytes into free fatty acids (FA) (45). Prostaglandin (PE) secretion is another common feature of neoplastic cells. PE supports MDSCs recruitment and activation as well as TAM polarization toward the M2 phenotype (46). Therefore, lipid metabolic reprogramming can be another important target for anti-cancer therapy.

### Molecular determinants of metabolic reprogramming: role of signaling pathways in driving responses to anti-cancer therapies

**Figure 2:**
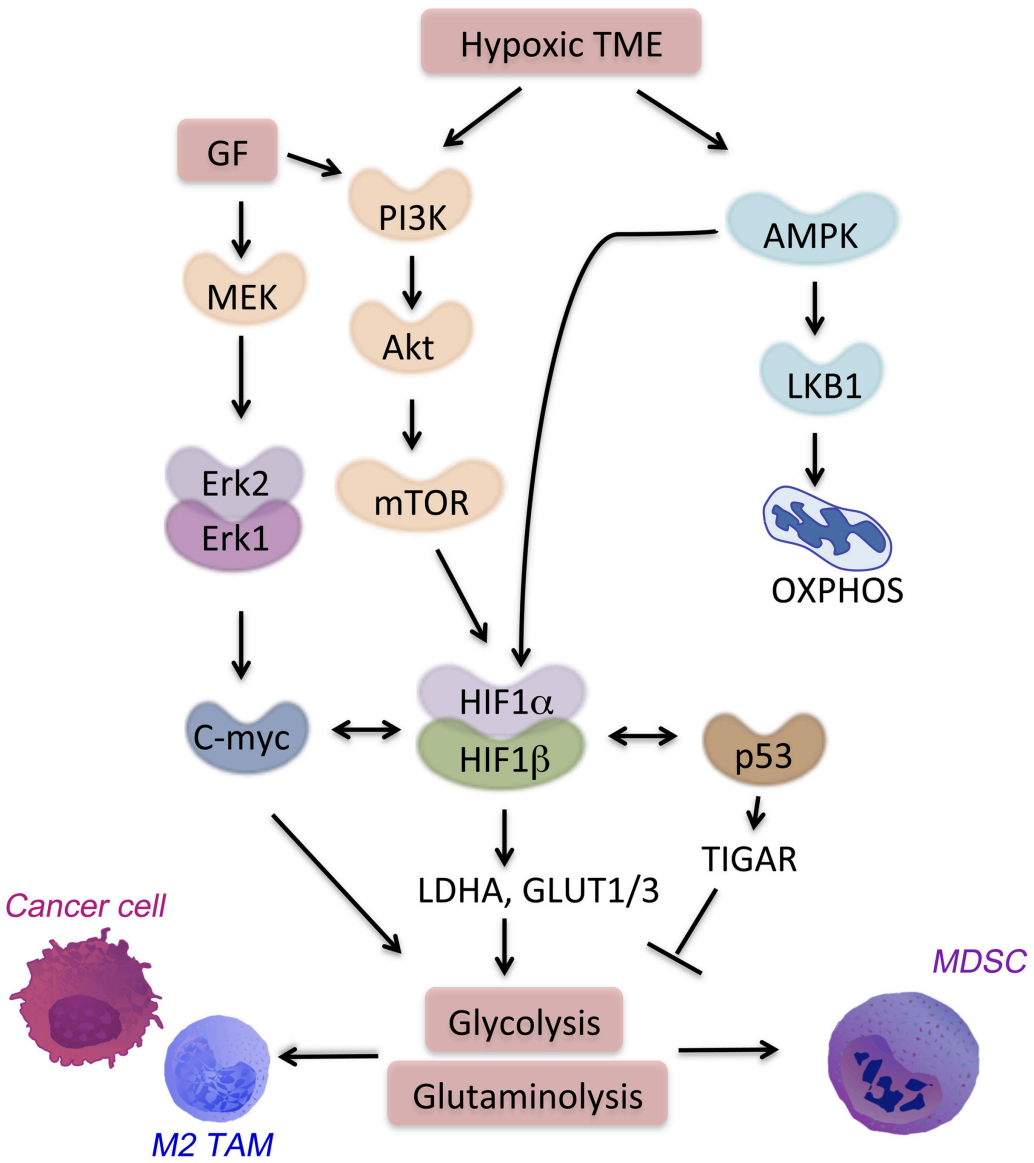
Signaling pathways in the hypoxic TME. HIF-1α is a major regulator of hypoxia-responsive genes in the TME. Multiple signaling pathways crosstalk with or activate HIF-1α. AMPK activation in the TME drives cell catabolism and is important for HIF-1α-regulated transcription. AMPK allows for metabolic adaptation in hypoxia with low ATP and lack of nutrient. Hypoxia also induces PI3K signaling through Akt-mTOR that promotes anti-apoptotic responses and metabolic shift toward glycolysis.

Many cancer and immune cell-derived factors present in the tumor microenvironment modulate neighbor cell metabolism. We will discuss here examples of molecular signaling pathways and transcription factors leading to aberrant metabolism in cancer and immune cells in the TME (Figure 2). All these signaling pathways are targets of hypoxia or feed into the metabolic pathways associated with hypoxia and cell starvation.

#### PI3K-AKT and mTOR pathways

PI3K-AKT pathway regulates both pro-inflammatory and immunosuppressive responses, thus drives the outcome of the disease (47). The signaling through PI3K-AKT leads to the activation of mammalian target of rapamycin (mTOR) (48) (Figure 2). A metabolic regulator that couples nutrient sensing to metabolic stress, mTOR regulates important tumor-related processes. mTOR activates HIF-1α. Together with other transcription factors (i.e., c-myc and Oct1), HIF-1α promotes expression of glycolytic genes such as hexokinase 2 (HK2), LDH-A, glucose transporter 1 (GLUT1) and 6-Phosphofructo-2-Kinase/Fructose-2,6-Biphosphatase 3 (PFKFB3) as well as PD-L1 (49). In CD8^+^ T cells, mTOR sustains glycolysis, their effector functions as well as cytokine-driven differentiation into Th1, Th2, or Th17 subsets (50).

It is now recognized, that PI3K-δ and γ isoforms play a key role in recruiting the inflammatory cells into the TME, increased angiogenesis and tumor growth (51). Moreover, tumor-derived chemo attractants that activate the PI3K-γ isoform lead to enhanced expression of α4β1 integrin. The integrins allow myeloid cell adhesion and infiltration into the TME. These myeloid cells later become immunosuppressive TAMs. Moreover, small-molecule inhibitors targeting PI3K δ and γ suppress this process and the associated tumor growth.

#### AMPK pathway

AMPK, a nutrient sensor, is a key regulator of oxidative phosphorylation as well as metabolic and oncogenic stress (52). AMPK suppresses anabolic pathways and activates cell catabolism. Activation of AMPK blocks cellular proliferation and tumor growth. However, in the absence of nutrients, loss of AMPK is not sufficient to promote cell proliferation. On the contrary, the loss of bioenergetics homeostasis leads to apoptosis of cancer cells that lack either AMPK or LKB1, a target of AMPK (Figure 2). AMPK regulates generation of CD8^+^ memory T cells, promotion of T regulatory cells, and suppression of T effector function (50, 53). The TME is characterized by enhanced hypoxia, nutrient deprivation and presence of anti-inflammatory cytokines (i.e., IL-10, IL-14, and TGF-β). In this niche, AMPK is activated in TAMs, MDSCs, and infiltrating T cells. AMPK activation leads to a metabolic switch toward oxidative phosphorylation and immunosuppression. Metformin, an activator of AMPK, showed a clinical efficacy on suppressing tumor growth (54). Interestingly, metformin supports expansion and survival of long-lived T memory cells by inhibition of glycolysis and promotion of oxidative phosphorylation in TME (55, 56). Targeting of AMPK in both immune cells and tumor cells might be a strategy to achieve a strong anti-tumor response in the hypoxic niche.

#### Transcription factors: HIF-1α, c-myc, and p53

HIF-1α regulates several homeostatic functions of mammalian cells under hypoxia. The outcome of HIF-1α activation is different depends on the cell type and the downstream signaling. For instance, HIF-1α can directly promote tumor growth via binding to the promoters of the genes involved in glycolysis, such as glucose transporters (i.e., GLUT1, GLUT3) or enzymes (i.e., LDH-A) (57). Activation of HIF-1α blocks pyruvate dehydrogenase kinase (PDK) thereby suppresses TCA activity and facilitates glycolysis in cancer cells. HIF-1α can also drive inflammation, angiogenesis, and tissue remodeling in the TME. MDSCs under hypoxia suppress both antigen-specific and nonspecific T cells (58). Moreover, HIF-1α upregulation leads to increased expression of PD-L1 that amplifies MDSCs suppressive activity (20). The detailed role of hypoxia and HIF-1α will be discussed below.

Similarly to HIF-1α, c-myc modulates several metabolic pathways by regulating the expression of enzymes, such as LDH-A and pyruvate dehydrogenase kinase. However, c-myc in addition to driving glycolytic metabolism, and in contrast to HIF-1α activates mitochondria biogenesis. This is necessary for c-myc-driven generation of mitochondrial intermediates for synthesis of DNA, proteins and fatty acids. C-myc also regulates glutamine metabolism in cancer and stroma cells by inducing the expression of glutamine transporters (e.g., SLC5A1) and glutaminase 1 (GLS1, the initial enzyme of glutaminolysis) (59, 60). Fatty acid oxidation is another target of c-myc in subset of triple negative breast cancer tumors (61). In activated T cells, c-myc increases glycolysis, glutaminolysis, and polyamine synthesis (62). Importantly, alternative polarization of TAMs requires c-myc expression that directly regulates expression of CD209 among other genes associated with M2 polarization (63).

The tumor suppressor protein, p53, is mutated in most of cancer cells and affects glucose metabolism and hypoxic responses in the TME. In normal cells, p53 down-regulates GLUT1 and GLUT4 genes. In contrary, mutations in the DNA binding domain of p53 abrogate its inhibitory effect. This allows for high expression of glucose transporters and promotes glucose metabolism (62). Mutant p53 is linked to high expression of hexokinase 2 (HK2). HK2 is upregulated in many tumors and supports high rates of glucose catabolism (65). In normal cells, p53 suppresses the same genes by targeting of TP53-induced glycolysis and apoptosis regulator (TIGAR). TIGAR blocks glycolysis and blocks intracellular ROS levels (66). Moreover, p53 limits glutaminolysis. In response to DNA damage or oxidative stress, p53 promotes the expression of mitochondrial glutaminase (GLS2) (67). Interestingly, plasticity of TAMs is regulated by p53-regulated repression of c-myc expression (68), which leads to suppression of M2 polarization.

### Hypoxia and tumor microenvironment

HIFs, a family of transcription factors, sense and respond to hypoxia. HIF-1α and HIF-2 α are the most widely studied family members (69). The activity of HIFs is regulated by post-transcriptional modifications, which involve hydroxylation of their proline and asparagine residues via prolyl hydroxylases (70). In normoxia, HIF-1α subunits are prolyl hydroxylated and tagged for proteasome degradation. During hypoxia, the HIF-1α protein translocates to the nucleus where it binds HIF-1β and regulates the transcription of multiple genes that harbor hypoxia response elements (HRE) in their promoters. Activities of HIF-1α and HIF-2α lead to upregulation of glucose transporters and multiple enzymes of the glycolytic pathway (71). Hypoxia blocks proryl-hydroxylase (PDH), via activation of PDK and decreases TCA cycle as well as the flow of pyruvate to mitochondria. HIF-1α mediates the metabolic switch from oxidative phosphorylation to aerobic glycolysis in cancer cells and immune cells in the TME (72). HIF-1α regulates the Th17/Treg balance in favor of the Th17 phenotype (73). Under hypoxic conditions, tumor cells have greater resistance to T cell-mediated cytotoxicity, due to HIF-1α-dependent transcriptional upregulation of PD-L1 (74). Similarly, PD-L1 expression in MDSCs in response to hypoxia further limits T cells cytolytic activity (20, 74). Therapeutic strategies targeting HIF pathway in cancer and immune cells might be beneficial for anti-tumor immune response (75).

### Metabolic interplay in the TME

There is evidence that targeting of aberrant metabolism in cancer cells inhibits tumor growth in animal models (76–81). Recent studies have also reported that targeting metabolic pathways in immune cells rather than directly in cancer cells may improve anti-cancer treatment (6, 82). The interactions between tumor and its microenvironment are driven in part by diffusible metabolites. This metabolic interplay in the TME, will be discussed in next paragraph with respect to nutrient competition and metabolic byproducts such as lactic acid. Changes in both metabolic substrates and products regulate immune and cancer cells in the TME.

#### Lactic acid, as a key player in tumor and immune metabolism

LDH-A, an enzyme responsible for the conversion of pyruvate to lactic acid, is significantly upregulated in cancer cells and is the main source of lactic acid in the tumor milieu. Extracellular lactic acid supports tumor expansion and immune evasion (6). Lactate strongly inhibits proliferation and anti-cancer function of human cytotoxic T lymphocytes (CTLs) (83, 84). We have recently reported that induction of global LDH-A deletion resulted in blockade of tumorigenesis (80). The effect of LDH-A deficiency in myeloid immune cells results in the same effect in KRAS-driven lung carcinoma model (6). It has been suggested, that acidosis induces hypoxia, HIF1α and immune checkpoint inhibitors such as PD-L1. We have demonstrated that LDH-A deletion in myeloid cells boosts anti-tumor T cell immunity through induction of IL-17-producing CD8^+^ T (Tc17) cells, likely via suppression of the lactate-driven PDL-1 expression (6). Furthermore, tumor-derived lactate supports the accumulation of myeloid-derived suppressor cells (MDSCs) while suppressing the function of natural killer (NK) cells and T lymphocytes (85, 86).

Although a major impact of lactate on the TME is acidification, lactate can be oxidized and provide energy in response to nutrient depletion. Recent studies have clarified that low pH in the presence of high lactate promotes M2 macrophage polarization (16, 87). Interestingly, treatment with proton pump inhibitors, which regulate the pH of TME, significantly limited tumor expansion (88). These treatments also restored functionality of tumor infiltrating lymphocytes and increased the therapeutic efficacy of adoptive immunotherapy in pre-clinical models (89). In another recent preclinical study, the effect of increasing alkalinity in TME in the context immunotherapy has been investigated (90). The combination of anti-PD-1 antibodies with bicarbonate administration led to improved antitumor responses in animals. These studies highlight how possibly reducing tumor acidity results in increased infiltration of cytotoxic T cell resulting in enhanced immune response.

#### Tryptophan and glutamine metabolism as a target in the TME

Amino acid metabolism plays important roles in TME, similarly to glucose metabolism. L-Tryptophan is one of eight essential amino acids, which are required for protein synthesis and support important cellular activities, such as proliferation. It is, now established that the catabolism of the amino acid tryptophan (TRP), through the kynurenine signaling, maintains an immunosuppressive environment in cancer (91, 92). The breakdown of tryptophan is catalyzed by the rate limiting enzyme indoleamine 2,3-dioxygenase (IDO) and TRP-2,3-dioxygenase 2 (TDO). These enzymes play important roles in cancer immune escape (Figure 1). IDO is induced by hypoxia in dendritic cells or macrophages (93, 94). Inhibition of IDO results in rejection of allogenic fetuses (95), suggesting that tryptophan breakdown is necessary for maintaining immune tolerance. The enzymatic activity of indoleamine 2,3-dioxygenase (IDO) and TRP-2,3-dioxygenase 2 (TDO) results in depletion of TRP in the local microenvironment. This in turn induces an amino acid starvation response in T cells leading to cell cycle arrest and cell death (96, 97). Many preclinical studies have proposed and demonstrated enhance anti-immune response to a combination of anti PD-1 therapy with IDO inhibitors (98).

3-hydroxy-kynurenine (3-HK) (KYN) and kynurenic acid (KA) are regulatory metabolites by which IDO and TDO favor immune tolerance. These molecules accumulate in the TME and promote differentiation of T cells into forkhead box P3-positive (FOXP3^+^) Tregs (Figure 1). This process occurs through the activation of the transcription factor, aryl hydrocarbon receptor (AhR) (99, 100). Moreover, AhR signaling promotes effector T cell anergy. There is evidence that both enzymes are upregulated in several of the cancer types, electing the inhibition of their enzymatic activity as a new promising anti-cancer therapy.

The glutaminase gene, is characterized by two splice variants in human. Glutaminase coverts glutamine into glutamate and is a promising target for anti-cancer therapy (42, 43). The 6-diazo-5-oxo-L-norleucine (DON), a non-standard amino acid originally isolated from Streptomyces in a sample of Peruvian soil, was the first glutaminase inhibitor, as early as 1950. DON acts as a substrate analog for glutaminase, competing with glutamine for the binding of the active site of the enzyme and therefore reducing the glutamine catabolism. In 1954, Dion and colleagues hypothesized for the first time the use of DON as anti-tumor molecule (101). As of today, many inhibitors targeting glutamine metabolismhave been developed. This includes; CB-839, a selective orally bioavailable inhibitor of both glutaminase splice variants. CB-839 showed a potent anti-proliferative activity in a triple-negative breast cancer (TNBC) cell line (102) and has recently entered the Phase 1 clinical trial in combination with paclitaxel.

### Gut microbiota influence cancer metabolism and responses to therapy

Recent studies suggest a role of gut microbiota in responses to the anti-cancer drugs (103, 104). The host immune response, and the drug toxicity are modulated by the intestinal flora. In part, these effects depend on the type of metabolites released by the different types of bacteria. For instance, β-glucuronidase, a carbolytic enzyme produced by gut-microbiota metabolites, controls the levels of the bioactive form of the chemotherapeutic drug Irinotecan and thus its side effects in the digestive tract (105). Furthermore, the metabolic activity of the gut microbiota induces ROS in the stroma. High level of ROS, prone epithelial cells to cytotoxic effect of oxaliplan, which is widely used for treatment of gastrointestinal malignancies (104, 106).

Further, gut microbiome modulates responses to immunotherapies. Microbial metabolites including the short-chain fatty acids (SCFAs) (107) such as butyrate, propionate, and acetate influence anti-cancer therapy. Butyrate, a histone deacetylase inhibitor (HDACi) regulates gene expression directly in the colon (108, 109). Furthermore, butyrate can be used as an energy source by colon cells. In 2014, Belcheva and colleagues demonstrated that butyrate supports growth of human adenomatous polyposis in mice models (110). In contrary, Donohoe and colleagues have reported that dietary fiber increase the butyrate levels in the colonic lumen and inhibit colorectal tumorigenesis *in vivo* (111). High butyrate–producing microbiota mitigate graft-versus host disease (GVHD) after allogenic bone marrow transplantation (112) suggesting its immunosuppressive effects. These observations highlight a possible role of the gut microbiome and their metabolites as pivotal players in the development of anti-cancer therapies.

## Summary and future directions

A crosstalk between metabolism and immune regulation in the TME is the major focus of current studies in multiple laboratories. It is clear that the metabolic imbalance in cancer cells as well as stroma cells dictates survival of the tumor and immune cells, and controls resistance to anti-cancer therapy. The roles that the same metabolic enzymes play in tumor and immune cells warrant consideration of future therapies. Moreover, the nutrient composition and availability, acting directly on the cells in the TME or through the gut microbiota enzymes, can strongly drive the outcome of the treatment and disease progression. Based on current evidences in the literature, it is clear that metabolic modulation is considered as an innovative therapeutic approach. Moreover, metabolic targeting in combination with already approved checkpoint blockade treatments may provide novel ways to facilitate long-term remission or cure of cancer.

## Author contributions

All authors listed have made a substantial, direct and intellectual contribution to the work, and approved it for publication.

### Conflict of interest statement

The authors declare that the research was conducted in the absence of any commercial or financial relationships that could be construed as a potential conflict of interest.
